# Hepatic Vein Aneurysm: An Extremely Rare Case with Successful Embolization

**DOI:** 10.3400/avd.cr.20-00130

**Published:** 2020-12-25

**Authors:** Surasit Akkakrisee, Keerati Hongsakul, Tortrakoon Thongkan

**Affiliations:** 1 Division of Interventional Radiology, Department of Radiology, Faculty of Medicine, Prince of Songkla University, Hat Yai, Songkhla, Thailand; 2 Division of Hepatopancreatobiliary Surgery, Department of Surgery, Faculty of Medicine, Prince of Songkla University, Hat Yai, Songkhla, Thailand

**Keywords:** hepatic vein aneurysm, embolization, imaging

## Abstract

Hepatic vein aneurysm is an extremely rare case. The etiology of hepatic vein aneurysms is uncertain, and endovascular treatment of this condition has not been reported. We report the case of a 71-year-old woman with right upper abdominal pain who was diagnosed with hepatic vein aneurysm and was successfully treated with an endovascular technique.

## Introduction

Visceral venous aneurysm is a rare vascular abnormality, and the porto-venous system is the most common site of this type of aneurysm.^1)^ However, hepatic vein aneurysm is rarer than visceral venous aneurysm that only one case has been reported to date.^2)^ Most venous aneurysms are asymptomatic. Complicated or symptomatic aneurysms are usually treated with surgery.^3)^ In recent years, increased utilization of imaging studies and endovascular treatments has led to new options for treating diseases in the venous system. Herein, we report the case of a 71-year-old woman with a hepatic vein aneurysm who underwent endovascular treatment and briefly review the current state of knowledge about this vascular abnormality.

## Case Report

**Figure 1:**
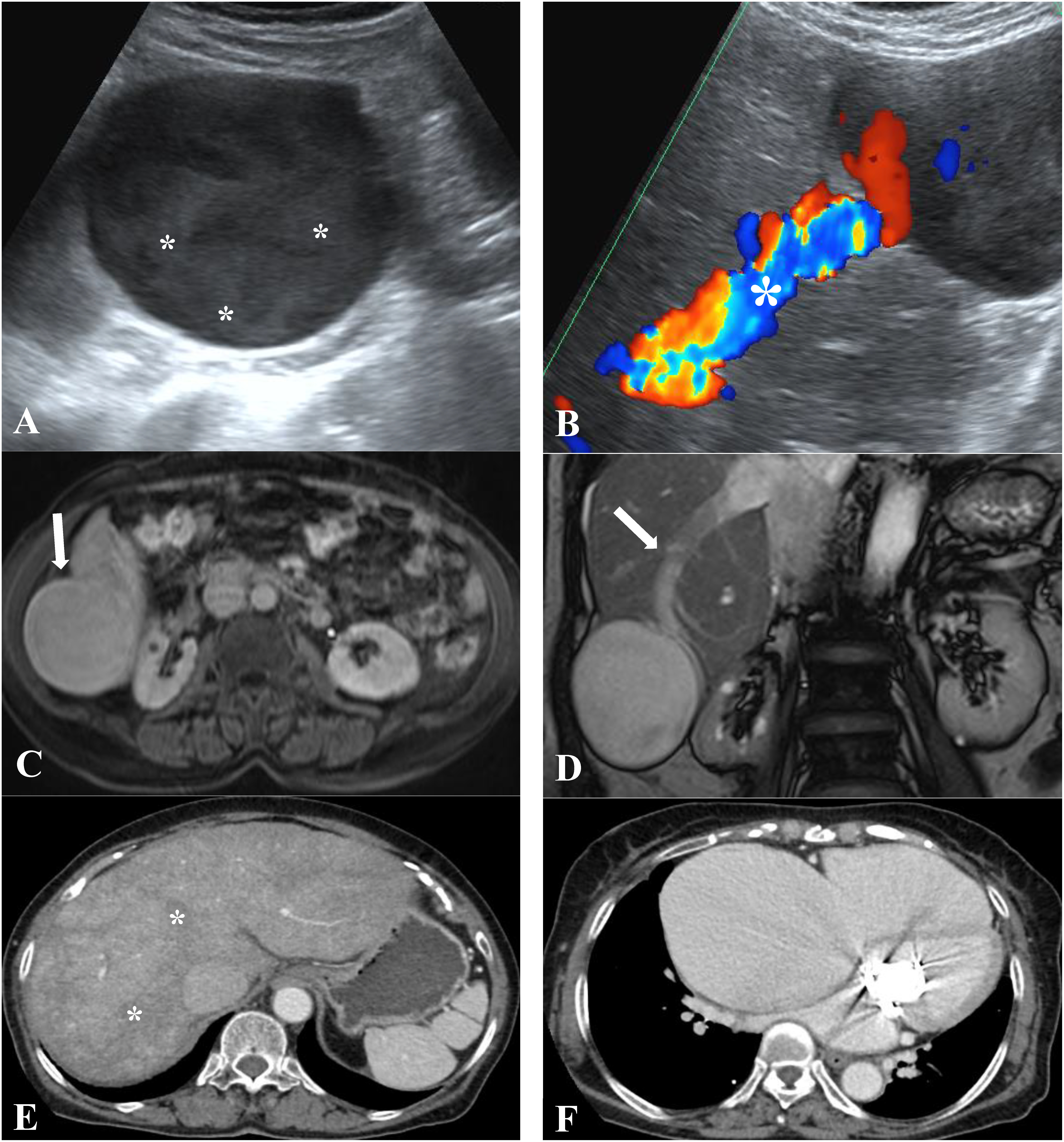
Fig. 1 (**A**) Grayscale ultrasound images delineate a hypoechoic lesion with suspected internal movable content (asterisks). (**B**) Color Doppler ultrasound images revealed a connection between the right hepatic vein (asterisk) and lesion. (**C**) Magnetic Resonance Imaging of the upper abdomen revealed a 6.4-cm ovoid-shaped exophytic lesion arising from hepatic segment 6 (arrow). The mass also showed delayed enhancement at 5 min after injection of gadoterate meglumine. (**D**) On the T2 coronal image, the connection between the hyperintensity lesion and right hepatic vein can be seen (arrow). (**E**) Porto-venous phase computed tomography images show a congested liver and poor enhancement of the hepatic veins (asterisks). (**F**) Cardiomegaly with markedly enlarged right atrium.

A 71-year-old woman, with a previous medical history of mitral valve replacement, presented with a complaint of right upper quadrant abdominal pain for 2 months. Her underlying diseases were congestive heart failure and hypertension. She had been taking warfarin since her valve replacement. At this presentation, physical assessment revealed hepatomegaly with mild tenderness at the right-sided abdomen, with no abnormal bowel sounds or stigmata of portal venous hypertension. Her baseline laboratory examinations revealed slightly increased alkaline phosphatase (approximately 157 μ/L) with normal bilirubin and other parameters of the liver function test. An ultrasound investigation revealed a large cystic lesion (approximately 6.4 cm in diameter) at the right hepatic lobe, and in this lesion, an internal vascular flow that connects it with the right hepatic vein was detected using color Doppler ultrasound (**Figs. 1A** and **1B**). At this time, a right hepatic vein aneurysm was suspected. A further magnetic resonance imaging (MRI) study of the upper abdomen confirmed a right hepatic vein aneurysm at hepatic segment 6 (**Figs. 1C** and **1D**). The diameter of the right hepatic vein was measured at 1.1 cm. After discussion with the surgeon, the patient was treated with embolization because of her comorbidities, which made her a poor candidate for surgery. Computed tomography (CT) angiography was performed for preoperative planning. A markedly enlarged right atrium and congested liver were depicted. No fistula between the hepatic vein and hepatic artery or portal vein was observed (**Figs. 1E** and **1F**).

**Figure 2:**
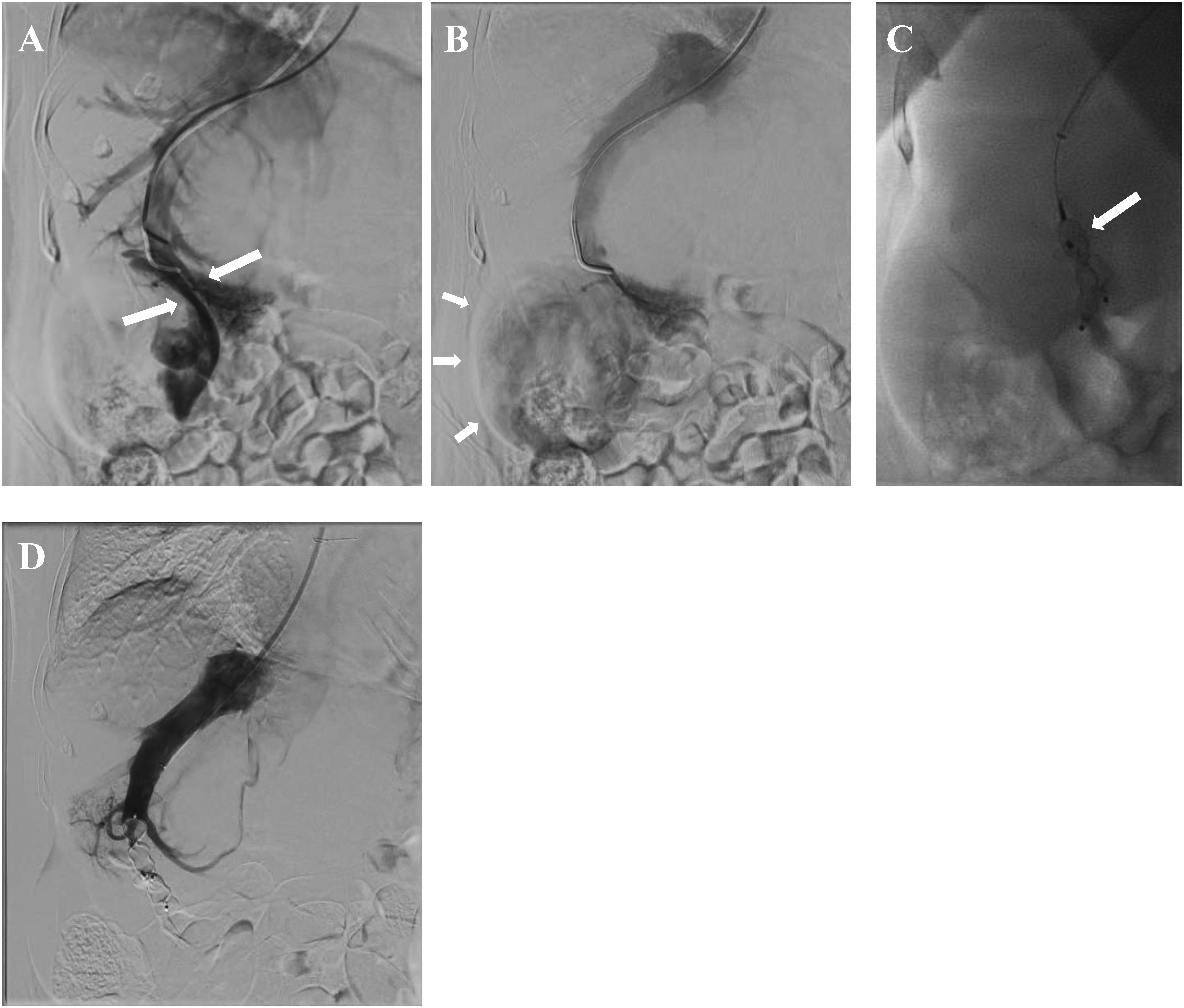
Fig. 2 (**A**) Selective right hepatic venogram revealed two draining veins (white arrows). (**B**) The contrast-filled aneurysm sac (arrows). (**C**) Two Amplatzer Vascular Plugs II were deployed (white arrow). (**D**) Post-embolization venogram revealed total occlusion of the draining veins.

**Figure 3:**
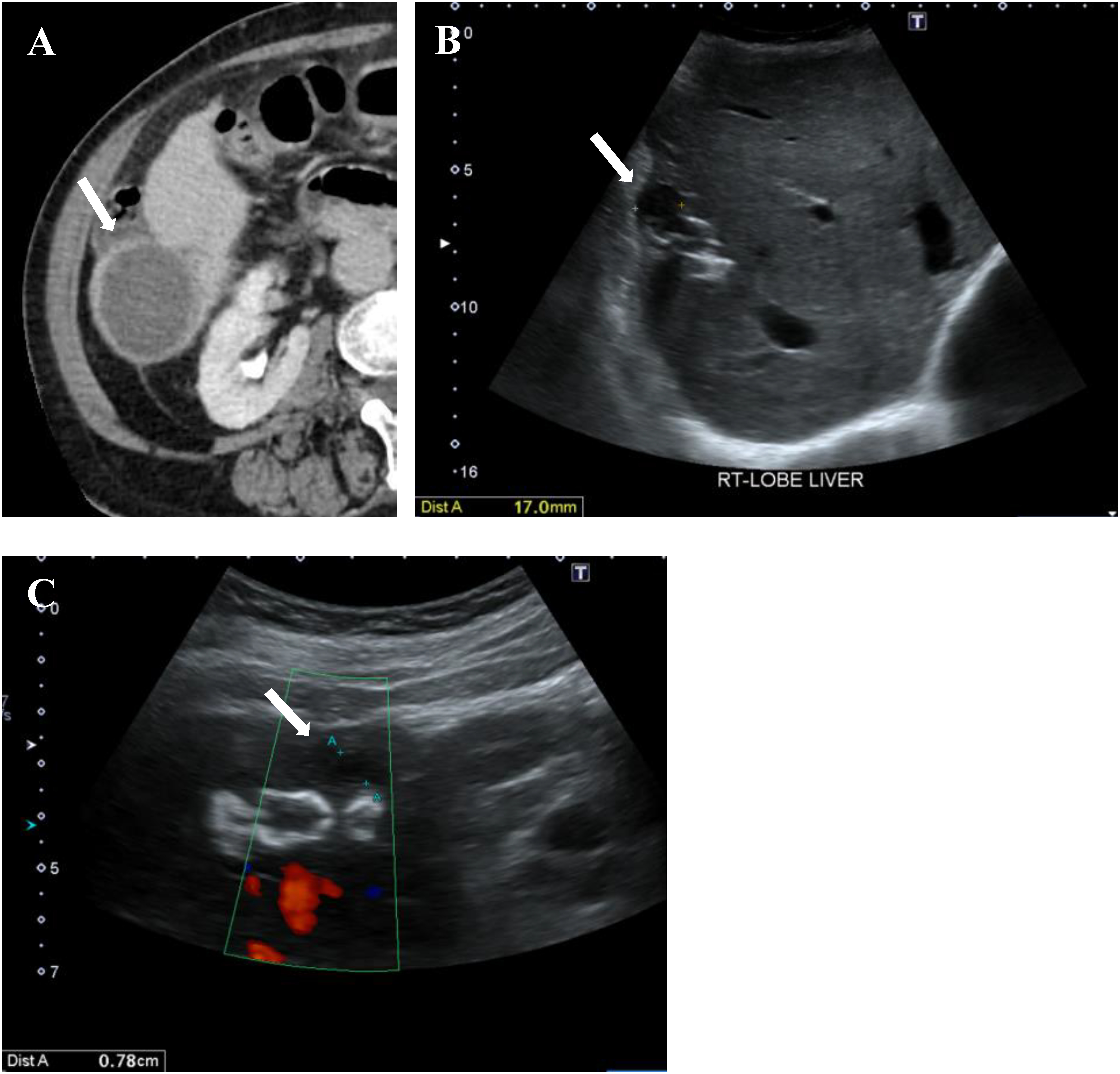
Fig. 3 Follow-up abdominal computed tomography at 4 months (**A**) and abdominal ultrasounds at 10 months (**B**) and 15 months (**C**) showing sequential decrease in size of the hepatic vein aneurysm without internal vascular flow (arrows).

A 6-French (Fr) vascular sheath was inserted via the right internal jugular vein over a 0.035-inch hydrophilic wire. The main right hepatic vein was selected with a 5-Fr Cobra catheter. A venogram via the Cobra catheter was acquired and revealed a large hepatic vein aneurysm, drained by two branches of the right hepatic vein. The prior 6-Fr vascular sheath was exchanged to a 6-Fr 45-cm-long Destination Guiding Sheath over a 0.035-inch stiff wire. The tip of the guiding sheath was located at the right hepatic vein. Two Amplatzer Vascular Plugs (AVP) II (12 mm×9 mm and 14 mm×10 mm in size, respectively) were deployed at the two draining veins of the right hepatic vein aneurysm. A post-embolization venogram showed complete occlusion of the draining veins (**Fig. 2**). There were no major procedure-related complications. Follow-up CT at 4 months (**Fig. 3A**) and ultrasounds at 10 and 15 months showed a markedly decreased size of the totally thrombosed right hepatic vein aneurysm (**Figs. 3B** and **3C**). The abdominal pain of the patient had subsided.

## Discussion

Visceral venous aneurysms are rare as compared with visceral arterial aneurysms. There have been many case reports of visceral venous aneurysms, but hepatic venous aneurysm is extremely rare, with only one case report published to our knowledge.^2)^ The etiology of visceral venous aneurysms is still debatable. It can be congenital or acquired. The acquired causes include liver disease, portal hypertension, trauma, inflammatory conditions, and connective tissue diseases.^1)^

Our patient had no history of abdominal trauma or surgery. Moreover, she had no clinical manifestation or history of connective tissue diseases or vasculitis. Our patient had clinical history of right-sided heart failure with the appearance of a markedly enlarged right atrium and a congested liver on CT images (**Fig. 1**). We presumed that the possible etiology of the hepatic venous aneurysm in our patient was the prolonged pressure in the venous system, similar with portal vein aneurysm, which is the prolonged pressure because of portal hypertension.^1)^ The prolonged right-sided heart failure from her underlying mitral valve insufficiency could have caused high back pressure to the inferior vena cava and hepatic venous system, resulting in aneurysmal dilatation of the hepatic vein. However, there has been no report of the association between congestive heart failure and venous aneurysm.

Imaging investigations are essential in diagnosing hepatic venous aneurysms and following up these patients. Tanaka et al. used ultrasonographic images of a hepatic venous aneurysm to demonstrate connection between the aneurysm and hepatic vein.^2)^ In grayscale ultrasound, hepatic venous aneurysms may appear similar to hepatic cysts. In our opinion, if turbulent flow is suspected within the hepatic cystic lesion, vascular aneurysm or pathology should be considered. Color Doppler ultrasound can help detect internal vascular flow in the aneurysm and its connection to the hepatic vein. CT and MRI are also useful for diagnosis and treatment planning. The appearance of the lesion in CT or MRI may be also similar to that of a hepatic cyst. Moreover, high central venous pressure may cause delayed enhancement of the hepatic veins and lesion. Therefore, delayed phase images are essential for confirming diagnosis (**Fig. 1**).

The management of visceral venous aneurysm is controversial. High-surgical-risk patients with asymptomatic small visceral venous aneurysms (<3 cm) can be observed and closely followed.^4,5)^ The indications for treatment of visceral venous aneurysm are the presence of symptoms or aneurysmal complications, such as thrombosis, rupture, or adjacent structure compression.^3)^ Surgery is the standard treatment for symptomatic aneurysms. The surgical technique of the procedure is dependent on the size and location of the aneurysm and presence of complications and comorbidities.^4–6)^ However, our patient was unsuitable for surgery because of her comorbidities.

Endovascular treatment is an alternative treatment for visceral venous aneurysms. Several studies have examined simple, safe, and minimally invasive endovascular treatments for visceral venous aneurysms.^7–9)^ Transjugular access is the appropriate route to access the right hepatic vein as it is parallel with the direction of the hepatic vein and most accessible site. Various embolic agents are available for embolization; in our patient, we used AVP to embolize the hepatic venous aneurysm because it is easy to use and can be retrieved and repositioned before deployment.^10)^ Pushable and interlocking coils can also be used for embolization in lesions of this type; however, coil dislodgment or nontarget embolization is a risk, and multiple coils may be needed for complete occlusion with increased cost of treatment. In this case, the use of glue and particles such as gelatin sponge or polyvinyl alcohol is unsuitable because they can cause nontarget embolization and serious complications such as pulmonary embolism.

## Conclusion

Hepatic venous aneurysm is an extremely rare visceral venous aneurysm with unclear etiology. Endovascular treatment can be an alternative treatment for symptomatic patients with high surgical risk.
